# The role and utility of population-based cancer registries in cervical cancer surveillance and control

**DOI:** 10.1016/j.ypmed.2020.106237

**Published:** 2021-03

**Authors:** Marion Piñeros, Mona Saraiya, Iacopo Baussano, Maxime Bonjour, Ann Chao, Freddie Bray

**Affiliations:** a Cancer Surveillance Section, International Agency for Research on Cancer, Lyon, France; b National Center for Chronic Disease Prevention and Health Promotion, Centers for Disease Control and Prevention, Atlanta, United States; c Infections and Cancer Epidemiology Group, International Agency for Research on Cancer, Lyon, France; d University “Claude Bernard” Lyon 1, Faculté de Médecine, Lyon, France; e Center for Global Health, National Cancer Institute, National Institutes of Health, Bethesda, MD, United States

**Keywords:** Cervical cancer, Registries, Global health, Epidemiology, Public health surveillance

## Abstract

Population–based cancer registries (PBCR) are vital to the assessment of the cancer burden and in monitoring and evaluating national progress in cervical cancer surveillance and control. Yet the level of their development in countries exhibiting the highest cervical cancer incidence rates is suboptimal, and requires considerable investment if they are to document the impact of WHO global initiative to eliminate cervical cancer as a public health problem. As a starting point we propose a comprehensive cancer surveillance framework, positioning PBCR in relation to other health information systems that are required across the cancer control continuum. The key concepts of PBCR are revisited and their role in providing a situation analysis of the scale and profile of the cancer-specific incidence and survival, and their evolution over time is illustrated with specific examples. Linking cervical cancer data to screening and immunization information systems enables the development of a comprehensive set of measures capable of assessing the short- and long-term achievements and impact of the initiative. Such data form the basis of national and global estimates of the cancer burden and permit comparisons across countries, facilitating decision-making or triggering cancer control action. The initiation and sustainable development of PBCR must be an early step in the scale-up of cervical cancer control activities as a means to ensure progress is successfully monitored and appraised.

## Highlights

• Cancer registries play a critical role in the Cervical Cancer Elimination Initiative.
• A framework facilitates understanding surveillance and monitoring for cancer control.
• Cancer registries provide short and long term indicators for cervical cancer control.
• There is great need to develop and sustain cancer registries in many countries.

## 1 Introduction

The World Health Organization (WHO) global strategy towards eliminating cervical cancer as a public health problem (World Health Organization, 2019a) seeks to scale up the implementation of evidence-based interventions for eliminating cervical cancer as a public health problem by meeting ambitious targets for human papillomavirus (HPV) vaccination, cervical cancer screening, and management of detected cervical disease. The WHO Initiative (World Health Organization, 2018a) places robust surveillance and monitoring systems at its heart, with population-based cancer registries (PBCR) playing a critical role in reporting the evolution of baseline cervical cancer incidence rates and the impact of interventions over time.

Worldwide there has been exponential growth in the number of PBCR in operation over the last half century: the first volume of *Cancer Incidence in Five Continents* (CI5) published in 1966, contained information from 32 registries in 29 countries (Parkin, 2006), whereas the latest volume published in 2017, included 343 PBCR in 65 countries (Bray et al., 2017). Yet, the pace of development has been much slower in low- and middle–income countries (LMIC) (Bray et al., 2017). Many of the highest estimated cervical cancer incidence and mortality rates are seen in countries and regions with limited surveillance at present, with only one in three LMIC having established high-quality PBCR.

In this article we aim to provide an overview of the critical role of PBCR in the WHO Initiative and in relation to other components of a comprehensive cancer control and surveillance program. We revisit some key definitions and, on the basis of selected examples, highlight the utility of PBCR in reporting local incidence and survival statistics, including cervical and other HPV-related cancers, and their potential role in enumerating precancerous lesions of the cervix. Given the paucity of recorded data, particularly in countries with the highest cervical cancer burden, we also highlight the relative value of estimates to trigger cancer control action. We further explore how PBCR data can be linked to screening and immunization databases to comprehensively assess the short- and long-term achievements and population impact of the WHO Initiative. Finally, we highlight the synergies between incidence and mortality, as well as several caveats, in the context of monitoring cervical cancer.

## 2 A framework for cervical cancer surveillance

**Fig. 1:**
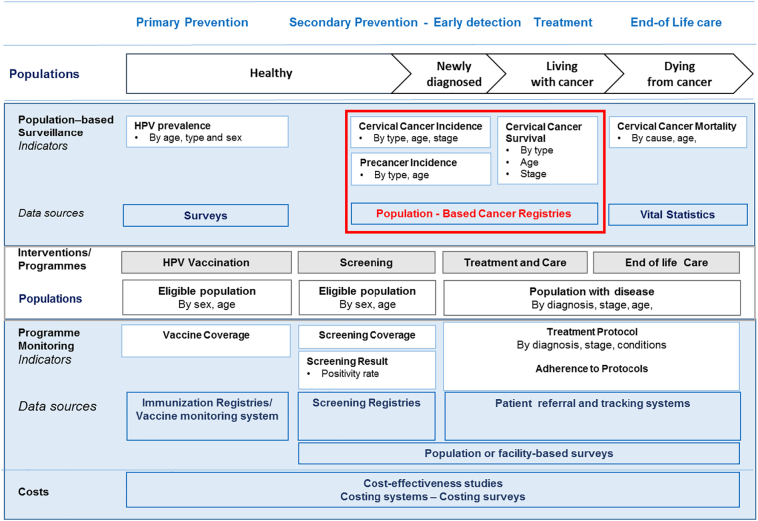
A framework for the surveillance and monitoring of a scaled-up cervical cancer control program, including the central role of PBCR.

Fig. 1 presents a comprehensive cancer surveillance framework that considers the populations and health information systems that span across the cancer control continuum. The different population subgroups targeted by specific interventions and pathways across multiple touchpoints in this continuum can be systematically recorded and tracked. The framework proposes a set of indicators obtained through routine population-based cancer surveillance as well as from a scaled-up implementation of cervical cancer control activities. At the population level, there are four key complementary measures: 1) the prevalence of specific risk factors for cervical cancer in the general population; 2) incidence (newly diagnosed cases of cervical cancer); 3) survival (percentage of patients surviving *n* years after the date of first diagnosis of cervical cancer) and 4) mortality (number of cervical cancer deaths). Two of these indicators, namely cancer-specific incidence and survival, are provided through PBCR.

Information on the prevalence of common risk factors for cancer (in general) or determinants of cervical cancer such as age, race/ethnicity, marital status and proxies of HPV exposure (e.g. age of initial sex or number of sexual partners) can be obtained through population-based surveys (Fig. 1), such as the WHO-developed STEPwise approach to surveillance (STEPS) survey, among others (DHS Program, n.d.; Viens et al., 2002; World Health Organization, 2018b). Information on the prevalence of cervical HPV infection by high-risk genotype is obtained through specific studies (de Sanjosé et al., 2007).

The ongoing assessment of the magnitude of the cervical cancer burden requires the use of three core population-level metrics, namely cancer incidence, cancer mortality and cancer survival (Fig. 1). Indeed, the ultimate measure of elimination of cervical cancer as a public health problem has been defined by WHO as the threshold incidence rate of 4 per 100,000 women (World Health Organization, 2019a), based on an assessment of incidence rates worldwide using data from PBCR. While HPV vaccination is a critical component of the WHO Initiative, its impact on the reduction of invasive cervical cancer incidence will take years to observe; the impact of cervical cancer screening on cancer incidence, survival, and mortality by stage will be seen more rapidly in populations covered by PBCR. Moreover, all cancer control interventions require monitoring through specific performance indicators and outcome measures, which can be subsequently linked to the information provided by PBCR (Fig. 1).

## 3 Population-based cancer registries: key definitions and concepts

PBCR are a system of continual data collection, storage, validation and analysis that permits the computation and dissemination of incidence rates and survival on all malignant neoplasms according to cancer type, stage at diagnosis, and other tumour and patient characteristics (Bray et al., 2014; Jensen and Storm, 1991; Parkin, 2008; Pineros et al., 2017a). As with any other public health surveillance strategy, information is disseminated and used for public health purposes, in this case, cervical cancer prevention and control. The recording and reporting of population-based cancer registry data are undertaken using international standards to ensure the comparability of the statistics generated from different PBCR. These have been developed and practiced over the last 50 years by PBCR through their professional organization, the International Association of Cancer Registries (IACR, http://www.iacr.com.fr).

While there are “specialized” registries that only collect data on specific cancer types or age groups, PBCR broadly collect information on all new cases of cancer occurring within a well-defined population and time period, and thus are able to generate incidence rates per 100,000 persons per year in the defined population-at-risk. To do this, PBCR usually require catchment populations that have access to diagnostic and treatment services in the defined area (Bray et al., 2014). The incidence rate provides an approximation of the average risk of developing a cancer, allowing geographic comparisons of the risk of disease in different populations at a given point in time (e.g. between or within countries, between ethnic groups) or temporal comparisons in one or more population across different time periods. When considering the impact of primary prevention strategies, a reduction in incidence is the appropriate statistic to use, requiring incidence by tumour site or an anatomic or histological subtype. While most PBCR attempt to collect data on stage of disease at diagnosis and the first course of treatment received, obtaining complete and comparable stage data across registry populations remains a challenge (Brierley et al., 2019).

The scale and profile of the cancer incidence burden in a population is critical to estimate health care infrastructure requirements, services and workforce requirements, and guiding resource allocation. With the revolution of accessible computing technology and electronic databases comes rapid reporting and data linkage, and with that an increasing demand for cancer data from the public health and clinical community. The core activities of PBCR have expanded beyond the provision of cancer statistics and many take on a more central role in the planning and evaluation of cancer control activities, including a more detailed evaluation of clinical care among cancer patients (Parkin, 2006; Siesling et al., 2015) as well as a range of registry-based epidemiologic research.

Cancer survival is a key metric of the effectiveness and quality of health care and cancer management systems (Coleman, 2014). It is calculated at the PBCR via the follow-up of vital status of reported cancer cases via passive means, at least where deaths can be effectively matched with close-to-complete civil registration and vital statistics (CRVS) data. In settings where CRVS data are either of insufficient quality or not available, active follow-up is needed, either indirectly (e.g. through close scrutiny of the medical records of patients) or directly (through contact with the individual patient or family member).

In terms of the geographic area covered by PBCR, while national coverage is desirable (and may be feasible in countries with relatively small populations), there is a careful balance between aspirations to build national cancer registration systems set against existing resources and the feasibility to sustain national operations producing high quality data. Government investments in building a high-quality subnational PBCR is likely to be more informative and of added value than a manifestly incomplete national system; ideally well-functioning subnational PBCR can be extended into a satellite system of PBCR over time increasingly representative of the national profile and thus supporting national and regional cancer policy (Bravo et al., 2018; Jedy-Agba et al., 2015; Koyande et al., 2016; Stillman et al., 2012).

## 4 The uses of PBCR to assess cervical cancer control: examples

### 4.1 Assessing incidence profiles

Observed cervical cancer incidence rates vary substantially across different populations. In the latest Volume of CI5 (Bray et al., 2017), age-standardized cervical cancer incidence rates per 100,000 women ranged from less than 2 in Riyadh, Saudi Arabia to over 85 in Harare, Zimbabwe for the diagnostic period 2008–2012. While these comparisons illustrate the striking global differences, local comparisons in incidence profiles among subnational registries within the same country, where available, permit further insight into the specific needs of at-risk subgroups. In Peru, for example, where a national cancer control plan prioritizing cervical cancer among others has been implemented, cervical cancer incidence rates in Arequipa are higher than in the capital, Lima (31.2 per 100,000 versus 22.2 per 100,000, respectively), while cervical cancer mortality rates are very similar in both areas (Pineros et al., 2017b).

**Fig. 2:**
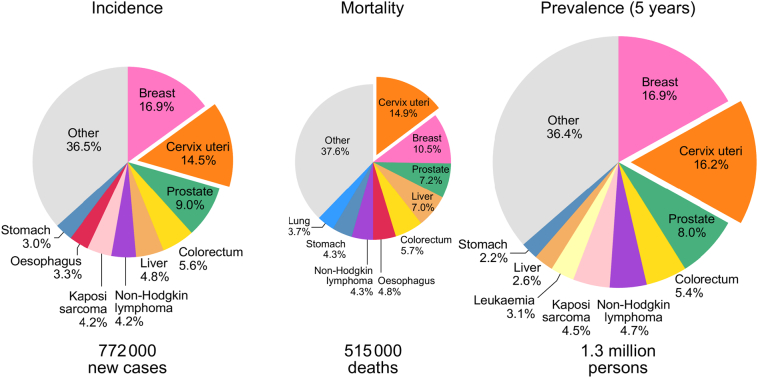
Distribution of main cancers in sub-Saharan Africa among females; incidence, prevalence and mortality in 2018.

Source: Globocan 2018 (Ferlay et al., 2018b).

To facilitate assessments of the burden of cervical cancer worldwide, the recorded data from PBCR are fundamental in building up global and national estimates. According to IARC's GLOBOCAN database (Stillman et al., 2012), an estimated 570,000 new cervical cancer cases are diagnosed each year (in 2018), representing 3.2% of all cancers among women globally. However, as incidence and mortality of cervical cancer are clearly linked to national levels of socioeconomic development – a proxy of underlying HPV-related risk factors and the availability and access to cervical cancer screening and curative treatment in a given country – the disease patterns vary considerably between and within world regions (Arbyn et al., 2020; Ginsburg et al., 2017). More than 85% of all cervical cancer cases globally occur in LMIC, with certain sub-Saharan African countries exhibiting estimated incidence rates ten times greater than the WHO-defined elimination incidence threshold of 4 per 100,000 (Bray et al., 2017; Egue et al., 2019; Lorenzoni et al., 2020). Cervical cancers account for 15% of new cancers reported among women in sub-Saharan Africa, with similar proportions observed for incidence, mortality and prevalence (Fig. 2).

The observed and estimated age-specific incidence rates also highlight important differences in the underlying risk patterns and the impact of interventions. In higher-resource countries, estimated cervical cancer age-specific incidence rates start rising after the age of 25 years, reaching a peak around the age of 40, whereas in lower-resource countries, rates continue to rise up to the ages of 55 to 69 years (Arbyn et al., 2020).

On a global scale there were an estimated 160,000, predominantly oropharyngeal and anogenital, cancer cases attributable to HPV infection in 2018 (de Martel et al., 2020). PBCR thus provide important information on the burden of these HPV-related cancers and enable their monitoring in relation to national scale-up of the HPV vaccination.

### 4.2 Assessing time trends

**Fig. 3:**
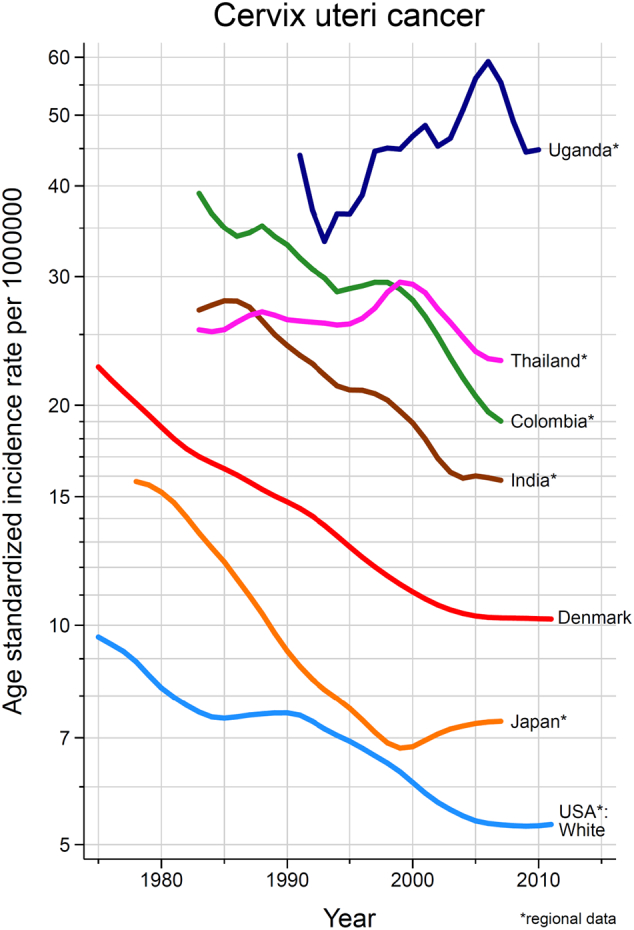
Trends in cervical cancer incidence rates, selected registries/countries, circa 1975–2012.

Source: CI5 –plus (Ferlay et al., 2018a).

**Fig. 4:**
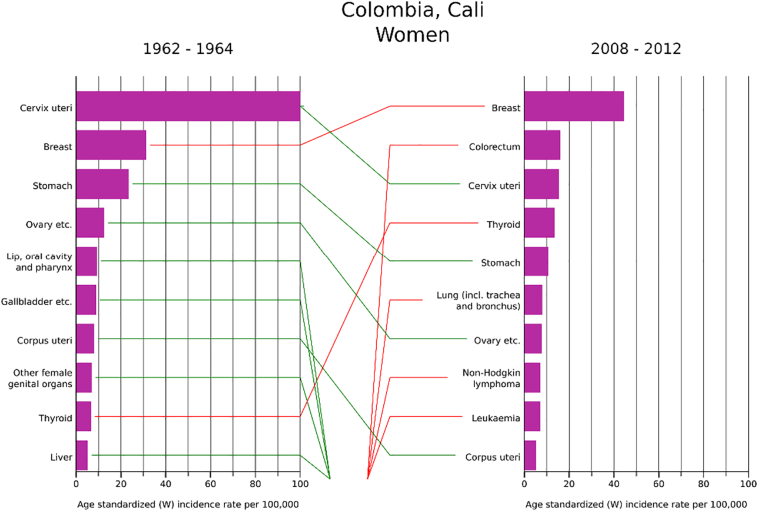
Changes in the distribution of common cancers across two diagnostic periods from 1962 to 2012, Cali Cancer Registry, Colombia.

Source: CI5 –plus (Ferlay et al., 2018a).

Data from longstanding PBCR enable the comparison of cervical cancer incidence rate changes over time, permitting evaluation of the evolution of risk against the impact of HPV vaccination and cervical cancer screening interventions. Fig. 3 shows trends in cervical cancer incidence rates in selected registry populations, clearly illustrating that while rates differ by an order of magnitude among populations, the trends are largely in decline. There are important exceptions to this temporal pattern, such as in Kampala, Uganda, one of the few longstanding sub-Saharan African PBCR included in CI5 (Ferlay et al., 2018a). Indeed, a recent study of trends, examined over a period of 10–25 years in 10 PBCR in eight sub-Saharan Africa countries, confirms a uniformity in the increasing incidence rates in the region other than in Mauritius (Jedy-Agba et al., 2020). Such observations may partially reflect variations in registration activity, but likely also relate to ongoing generational changes in risk due to changing sexual behaviour and exposure to high-risk HPV types. In contrast, in populations “exposed” to effective screening services, these cohort effects are countered by changes across age groups at a given point in time, so-called period effects. Longstanding PBCR such as in Cali, Colombia, permit the observation of changing patterns in cancers between time periods, as illustrated in Fig. 4, where cervical cancer ranked first among women in 1962–66 and ranked third by 2008–2012. If data on tumour stage are available, PBCR can be used to examine down-staging in cervical cancer rates as a result of screening implementation.

### 4.3 Benchmarking survival

Population-based measures of cancer survival relate to the effectiveness and quality of health care and cancer management systems (Coleman, 2014), and thus benchmarking reported cancer survival between registries is a powerful tool to identify differences between populations and mobilize governments to investigate shortcomings and take specific actions to improve clinical care. International benchmarking initiatives on cancer survival involving PBCR worldwide include IARC's SURVCAN-1 and -2 studies (Sankaranarayanan et al., 1998; Sankaranarayanan et al., 2011), the CONCORD-studies (Allemani et al., 2018; Allemani et al., 2015) and the more recent IARC-led SURVMARK project (Arnold et al., 2019).

In CONCORD-2, five-year cervical cancer survival (based on cancers diagnosed in 2005–2009) showed significant variation among countries, from below 60% in 20 countries to over 70% in seven countries (Ginsburg et al., 2017). In the US, cervical cancer survival data, from PBCR (covering 80% of the population) for the period 2001–2009, showed persistent differences in survival between black and white women in all US states included in the study (Arnold et al., 2019). The underlying and consistently higher proportion of cancers diagnosed at a distant stage among black women indicated the need for specific targeted interventions to improve survival among this population (Benard et al., 2017). Similar results by ethnicity have been reported by the South African National Cancer Registry where higher 5-year survival observed among whites and Indians/Asians (60–80%) was reported relative to blacks and coloureds (40–50%) (Olorunfemi et al., 2018). More recently, cervical cancer survival data compiled from 11 sub-Saharan PBCR reported an average 3- and 5- year relative survival of 44% and 33%, respectively, with two-thirds of staged cases diagnosed at late stages, III-IV (Sengayi-Muchengeti et al., 2020).

Benchmarking cancer survival studies in LMIC (SURVCAN-1 and -2) have been published as IARC reports (Sankaranarayanan et al., 1998; Sankaranarayanan et al., 2011); a third iteration of these studies (SURVCAN-3) is assessing survival data from 86 PBCR in 36 countries (https://survival.iarc.fr/Survcan/en/). A key aim of the project is to support registries to develop their own capacity to collect and analyse survival data.

### 4.4 Differentiating estimated and observed incidence

IARC, as part of its mandate as the cancer agency of WHO, provides estimates of the national cancer burden in 185 countries worldwide, currently for the year 2018. These estimates are based on observed numerator data on incidence (from PBCR), mortality (from CRVS), and population denominators (from population censuses), using transparent and reproducible methods that are described elsewhere (Ferlay et al., 2019). Cancer incidence estimates provide a means to assess the national profile and burden compared to other countries within world regions for the purposes of prioritizing cancer control activities. In countries with large populations that have one or more subnational PBCR, estimates may also be computed for subnational administrative areas, as undertaken, for example, in Brazil (Ministerio da Saude, Instituto Nacional de Cancer Jose Alencar Gomes da Silva, 2020). While useful, national and subnational estimates cannot substitute the observed cancer incidence data provided and continuously updated by PBCR. Estimates provide a useful snapshot of the disease magnitude but cannot accurately convey the temporal evolution of cancer-specific incidence or mortality rates based on observed data collected locally and continously over time.

### 4.5 Monitoring the long-term impact of scale-up of activities - how far are countries from the elimination target?

**Fig. 5:**
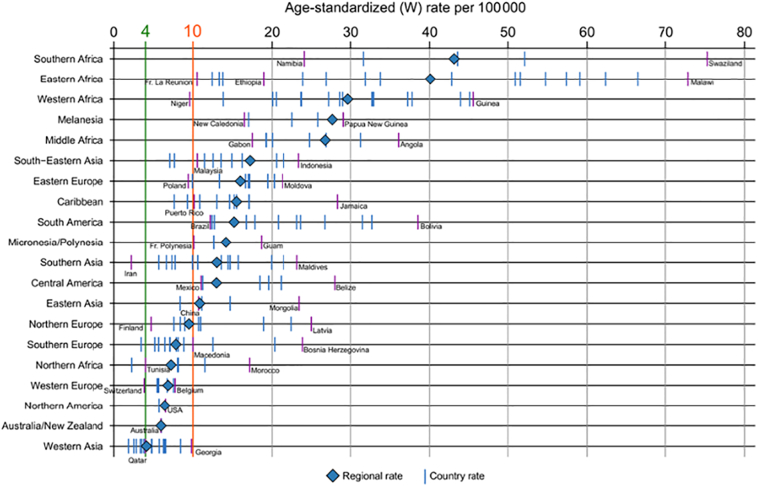
Estimated cervical cancer age standardized incidence rates (per 100,000) in 2018 by world region and individual countries (countries with minimum and maximum rates within region are labelled; the green line indicates the cervical cancer incidence rate threshold of 4 per 100,000 defined by the WHO Cervical Cancer Elimination Initiative). (For interpretation of the references to colour in this figure legend, the reader is referred to the web version of this article.)

Source: Globocan 2018 (Ferlay et al., 2018b).

Documenting whether and when countries attain the defined cervical cancer elimination target incidence rate requires long-term monitoring, particularly in countries with currently high incidence rates; it has been estimated that, even with rapid scale-up to reach high-coverage of screening combined with HPV vaccination from 2020 onwards, the threshold incidence rate in LMIC would take some 50–80 years to be met (Simms et al., 2019). Despite the great value of such modelling exercises in estimating progress towards the target, the long-term nature of the enterprise may potentially discourage decision-makers wishing to demonstrate favourable results in a shorter timeframe. Investing in PBCR to monitor cervical cancer incidence rates, alongside intermediate outcomes and program performance measures included in Fig. 1 provides a platform from which governments and policy makers can demonstrate the ongoing impact of the scale-up of interventions, including lower cervical cancer incidence, down-staging the invasive cancers diagnosed, longer survival after diagnosis, and more lives saved. Fig. 5 depicts national estimates of cervical cancer incidence rates by sub-region and an indication of the specific situation in different countries. The global cervical cancer estimates available at IARCs Cancer Today website (Ferlay et al., 2018b), can be used to reproduce Fig. 5 focusing in greater detail on particular regions of the world, as has been done for countries in Latin America (Pilleron et al., 2020) to provide useful information for regional stakeholders tasked with supporting the WHO Initiative at a regional level (World Health Organization, 2019a).

Beyond monitoring the magnitude of the cervical cancer burden, there is an overwhelming need to develop sustainable PBCR to monitor and measure the impact of *all* cancer control actions - including tobacco control, the early detection and screening (where feasible) of breast and colorectal cancers, and the vaccination of neonates against hepatitis B infection (Parkin, 2008).

### 4.6 Estimating the number of cervical cancer cases that could be averted through HPV vaccination

**Fig. 6:**
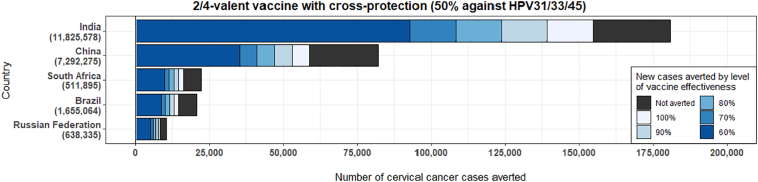
Estimated number of cervical cancer cases averted by HPV vaccination of a cohort of girls (age 15 years), by level of vaccine.

Source: Estimates by co-authors (IB and MB, not yet published).

Stimulating public health action using surveillance data is an important task in the context of the cervical cancer Initiative, as illustrated in the following example. Cervical cancer incidence from PBCR can be used to estimate the number of cervical cancer cases that would be averted (in a lifetime) for a given cohort of vaccinated girls according to varying scenarios of vaccine effectiveness according to the number of HPV types included in the vaccine used and the reduction in population-level prevalence of high-risk HPV types. Fig. 6 illustrates estimates of the lifetime number of cases averted with the bivalent vaccine targeted to HPV types 16 and 18 (that provides cross-protection against HPV types 31/33/35 (Malagón et al., 2012)), among girls aged 15 years in 2018 in the BRICS (Brazil, the Russian Federation, India, China and South Africa). The expected cohort-specific burden of cervical cancer in the absence of vaccination is presented, according to differing levels of vaccine effectiveness, dependent on the postulated success of the vaccination program and the type of HPV vaccine used. A total of 322,000 cervical cancer cases could be expected in the BRICS combined in the specific cohort. Of these cases, it is estimated that HPV vaccination could avert between 154,000 to 257,000 cases. The expected cohort-specific burden of cervical cancer in the absence of vaccination has been estimated by combining age-specific incidence rate projections from GLOBOCAN estimates (Ferlay et al., 2019) and cohort-specific mortality rates by age as reported by the United Nations (United Nations, Department of Economic and Social Affairs, 2019). The expected impact of HPV vaccination was estimated by combining different levels of vaccination effectiveness with fractions of cervical cancer attributable to various high-risk HPV types within the populations (Basu et al., 2011; Guan et al., 2012; Serrano et al., 2014).

Cohort-specific projections of the cervical cancer burden, using PBCR data, help to assess the impact of HPV vaccination among today's young girls; therefore, they provide an early perspective on the expected public health return on investment of HPV vaccination. These projections can be monitored and periodically validated and updated through the assessment of HPV prevalence by type in the population using cross-sectional HPV prevalence surveys (Franceschi et al., 2018).

### 4.7 Monitoring the short-term impact of scale-up of interventions: precancerous conditions of the cervix

In addition to cancer incidence, PBCR can evaluate and monitor more immediate outcomes of cervical cancer control interventions, providing information on specific precancerous conditions, where there is evidence that they are associated with persistent high-risk HPV infection. This is the case for cervical intraepithelial neoplasia grades 2 and 3 (CIN2/3), vulvar intraepithelial neoplasia grades 2 and 3 (VIN2/3), vaginal intraepithelial neoplasia grades 2 and 3 (VaIN2/3) and anal intraepithelial neoplasia (AIN) (De Vuyst et al., 2009; van de Nieuwenhof et al., 2008). If cases of precancers are to be collected by the PBCR, previous research suggest giving priority to lesions that have greater likelihood of progression and a higher percentage of persistent HPV infections leading to malignancy - such as grade 3 lesions, often considered synonymous with *in situ* cancer (Saraiya et al., 2008).

The systematic collection of precancerous lesions of the uterine cervix by PBCR is not widely practiced, as the number of pre-invasive lesions significantly outweighs that of invasive cancers. In the US, where recording of CIS/CIN-3 began in 1986, registries discontinued such practices 10 years later as a result of the additional work load and the inability to capture all pre-invasive lesions diagnosed in different clinical settings (Saraiya et al., 2015). The introduction of HPV vaccination in 2006 motivated the US Centers for Disease Control and Prevention (CDC) to set up a sentinel registry-based project in 2008 to monitor the impact of HPV vaccination on reported pre-invasive lesions (Hariri et al., 2012). Furthermore, monitoring pre-invasive lesions is important for determining the burden of a preventable disease, identifying effects of vaccination on future diagnoses and developing targeted programs. After a pilot feasibility study, surveillance of CIN 3 and adenocarcinoma in situ (AIS) is now performed routinely in four PBCR of the US network of registries (Flagg et al., 2014; Watson et al., 2017).

Although the impact of vaccination on HPV-associated high-grade cervical lesions is expected to take longer to be observed relative to other indicators (e.g. the prevalence of HPV infection and genital warts), surveillance data have shown that an effect of HPV vaccination may already be seen 3–5 years after vaccination. For example, in the US, where HPV vaccination has been recommended since 2006 among girls, a significant decline in the rate of CIN2+ (per 100,000) in women aged 18–24 years was observed over the period 2008–2016 (McClung et al., 2019). The authors attributed the decline to implementation of updated screening recommendations as well as to the impact of HPV vaccination (McClung et al., 2019). Similar results have been reported in an ecological study using data from the Victoria Cervical Cancer Register in Australia, where reductions in incidence of high-grade cervical lesions in girls aged <18 years were observed within three years of vaccine introduction (Brotherton et al., 2011).

The Nordic countries provide illustrative examples of how comprehensive surveillance through PBCR and linkages can quantify the impact of HPV vaccination and cervical cancer screening (Enerly et al., 2012; Kjaer et al., 2018; Nygard et al., 2014; Thamsborg et al., 2018). In the US, the information systems of the immunization program are being linked with PBCR to examine incidence of cervical cancer precursors in the HPV immunization-eligible female population (Potter et al., 2015). These examples demonstrate the usefulness and value of cancer surveillance systems to measure the impact of interventions. They also highlight how governments can invest in information systems to systematically record and link data on individual women from HPV vaccination clinics, cervical cancer screening services, hospitals, as well as PBCR and CRVS. In countries where these programs and interventions are being developed, it is critical to ensure overlap in geographic and population coverage to maximize the likelihood of data linkage and measurement of impact.

## 5 PBCR and relation to other data sources

### 5.1 PBCR and mortality data

Alongside hospital discharge records and pathology reports, death certificates (with specification of underlying causes of death) are an important data source in ensuring completeness of the PBCR and, where follow-up and linkage of cancer and death registries are in place, they provide critical information on the vital status of registered cancer cases for survival analysis (Bray et al., 2014). Despite the well-documented benefits of CRVS systems and the essential data they provide, e.g. in assessing whether UN Member States are on track to meet the Sustainable Development Goal health targets (World Health Organization, n.d.), many countries do not have adequate systems in place (Mikkelsen et al., 2015; Phillips et al., 2015). It is estimated that cause of death data are complete and of high quality in only one in four WHO Member States (World Health Organization, 2018c). Similar to the availability of PBCR, the deficits occur predominantly in LMICs, coinciding with the paucity of PBCR, and often where the cervical cancer incidence and mortality burden is high. As an illustration, the WHO African and South-East Asia regions have a high cervical cancer burden but a completeness of national mortality data estimated at 6% and 10%, respectively (World Health Organization, 2018c). Moreover, where there are functioning PBCR, mortality data are often not available or accessible to the PBCR; the lack of this critical source of case-finding can lead to under-estimation of cancer incidence in these areas.

In the framework of the WHO Initiative, cervical cancer mortality rates and their trends are of direct relevance to monitoring the long-term effectiveness and impact of screening programs (World Health Organization, 2019a). In strengthening health information systems and vital statistics, two particular concerns emerge regarding cervical cancer mortality data. The first is related to the quality of the cause(s) of death information captured in death certificates, and the training of health care professionals to ensure the systematic and accurate recording and coding of causes of death. Thus far, the results obtained by several training initiatives in LMIC to improve the quality of cause of death information in death certificates are encouraging (Hart et al., 2020; Hazard et al., 2017; Miki et al., 2018; Uddin et al., 2019). Teaching the importance and completion of death certificates with accurate causes of death is an area for improvement in medical schools globally, not only in LMIC (Lakkireddy et al., 2004; Menyah and Bohra, 2016; Rolfe et al., 2001).

The second aspect refers to methodological concerns regarding the use of cervical cancer mortality rates to assess time trends where there is substantial misclassification of cancer cause of death. Deaths specified as resulting from ill-defined uterine cancers (ICD-10 C55) represent a significant percentage of all uterus-related deaths in many countries and can lead to a misleading interpretation of the corresponding time trends. Analysing time trends in mortality from cancers of the cervix and corpus uteri requires an assessment of the number of deaths either unspecified as “uterus, unspecified site” (ICD-10 C55) or combined as unspecified and corpus uteri cancer deaths (Loos et al., 2004), as well as the potential change in coding over time. To avoid misleading interpretations, it is often necessary to either apply a reallocation of unspecified causes of death or simply focus analyses on women aged 50 years or younger, where unspecified deaths are more likely to be from cervical cancer. Reallocation methods should be integrated systematically into the analysis of cervical cancer mortality (Pilleron et al., 2020; Loos et al., 2004).

### 5.2 Use of PBCRs to measure the impact of cervical cancer screening

In countries with organized cervical cancer screening, a central screening registry can capture individual data with details of the screening process (from invitation to screening, attending screening, receiving screening results, referral to subsequent diagnoses and treatment of detected lesions, through to recall to the next screening). In Europe, many countries have organized cervical cancer screening programs and associated screening registries with personal identifiable data to enable linkage with PBCR (Elfstrom et al., 2015). This facilitates the collection of the key indicators required, providing response rates and screening coverage in the population, as well as estimating the proportion of women diagnosed with CIN2+ or other screening outcomes. The coverage of screening obtained via screening registries can be easily contrasted and linked with cancer incidence data from PBCR, as has been done in Sweden (Elfstrom et al., 2016), permitting assessment of the effectiveness and impact of the screening program at amore granular administrative district level.

Linkages between screening registries and PBCR facilitate the collection and computation of additional performance indicators, including cervical cancer incidence rates in unscreened (and under-screened) populations, the proportion of those with normal or abnormal cytology, and an estimation of the false-negative rate of the screening regimen used. The importance and utility of these indicators for cervical cancer control has led to a European call for greater engagement between screening programs and PBCR (Anttila et al., 2015), particularly as linkages are feasible in many countries in Europe (Majek et al., 2019). However, the situation is more complex in settings with more fragmented health care systems where opportunistic screening is the norm, and many different actors offer and/or perform screening without adequate follow-up or referral (Murillo et al., 2008). Often screening programs are launched in areas not covered by PBCR, making linkage of screening and PBCR data impossible. Screening coverage is the number of eligible women screened in a population, divided by the number of women eligible for cervical cancer screening in the population, as defined by eligibility criteria. Nonetheless, there may exist the tendency to define screening coverage as the totality of screens undertaken in health centres over the total number of screens allocated to those health centres, following a “quota” scheme that precludes the reporting of important indicators.

Over the last decades, estimates of cervical cancer screening coverage have come from population-based national demography and health surveys or behavioural risk factor surveillance surveys (Wang et al., 2015). In many LMIC, surveys providing this information have been supported by national governments and the US Agency for International Development (Viens et al., 2002). Despite concerns of bias due to self-reporting and the non-standardisation of definitions and measurement methods, surveys may be the only available data source on cervical cancer screening coverage in some countries, at least until screening registries are developed and function as integral components of health surveillance systems (Chao and Sivaram, 2018). Conscious of these limitations, WHO and other partners developed a toolkit for cervical cancer programs in 2019 to help standardize data collection and improve comparability (World Health Organization, 2019b). While the information collected using the toolkit can be compared with the magnitude of cervical cancer incidence, such an ecological approach is limited by a lack of representativeness of the surveys however, as well as the inevitable lag between survey results and the availability of cancer incidence data reported from PBCR. Consistency in the definition of time period of cancer diagnosis and stage of disease at diagnosis are particularly important in evaluating cancer screening efforts.

## 6 Conclusions

We have highlighted the importance and uses of population-based cancer registries in describing and understanding the local cancer situation, in planning national cancer control activities, and evaluating their impact generally and, specifically, in support of the WHO Initiative and associated planning at the national level. While the contribution of PBCR has increased as technology has advanced, the gaps between high- and low-resource countries appear to be widening. The development and sustainability of PBCR in countries with high cervical cancer incidence rates can notably be reinforced within the framework of the Initiative.

In supporting LMICs to develop PBCR and monitoring systems for cancer prevention interventions, we stress the importance of empowering countries to develop their own capacity to collect and analyse data. The process of strengthening PBCR is one example of a dual investment in local and global health planning (Shiffman and Shawar, 2020). The Global Initiative for Cancer Registry Development (GICR, http://gicr.iarc.fr), a multi-partner initiative led by the International Agency for Research on Cancer (IARC), works to reduce existing inequities in data availability. The GICR is committed to helping to initiate and strengthen PBCR in LMIC to ensure countries are informed by real data. PBCR are central in the planning and evaluation of the scale-up of HPV vaccination, cervical cancer screening and treatment as part of the WHO Initiative, and equally in documenting the impact of comprehensive cancer control policies aimed at reducing the burden and suffering from the disease.

## Disclaimers

Where authors are identified as personnel of the International Agency for Research on Cancer/World Health Organization, the authors alone are responsible for the views expressed in this article and they do not necessarily represent the decisions, policy or views of the International Agency for Research on Cancer/World Health Organization.

The findings and conclusions in this report are those of the authors and do not necessarily represent the official position of the U.S. Department of Health and Human Services, the Public Health Service, the Centers for Disease Control and Prevention, the National Cancer Institute, or the authors' affiliated institutions.

Use of trade names is for identification only and does not imply endorsement by the Public Health Service or by the U.S. Department of Health and Human Services.

## CRediT authorship contribution statement

Conceptualization of the paper: FB and MP; Fig. 1, Fig. 2, Fig. 3, Fig. 4, Fig. 5 have been conceptualized and elaborated by FB and MP and at the Section of Cancer Surveillance; Fig. 6 has been conceptualized and elaborated by IB and MB.

Writing review and editing: all authors have participated in writing, reviewing and editing.
